# ﻿A new species of *Typhonium* (Araceae) from Vietnam

**DOI:** 10.3897/phytokeys.238.112973

**Published:** 2024-02-16

**Authors:** Hong Truong Luu, Nga Nguyen-Phi, Quoc Dat Nguyen, Hieu Cuong Nguyen, Hong Thien Van, Xuan Bach Nguyen-Le

**Affiliations:** 1 Southern Institute of Ecology, Institute of Applied Materials Science, Vietnam Academy of Science and Technology, No. 1D, TL 29 Street, Thanh Loc Ward, District 12, Ho Chi Minh City, Vietnam Vietnam Academy of Science and Technology Ho Chi Minh City Vietnam; 2 Graduate University of Science and Technology, Vietnam Academy of Science and Technology, No. 18, Hoang Quoc Viet Street, Cau Giay District, Ho Chi Minh City, Vietnam Graduate University of Science and Technology Ho Chi Minh City Vietnam; 3 Department of Ecology and Evolutionary Biology, University of Science, Vietnam National University HCMC, 227 Nguyen Van Cu Street, District 5, Ho Chi Minh City, Vietnam University of Science Ho Chi Minh City Vietnam; 4 Vietnam National University – HCMC, Linh Trung Ward, Thu Duc City, Ho Chi Minh City, Vietnam Vietnam National University Ho Chi Minh City Vietnam; 5 Institute of Biotechnology and Food Technology, Industrial University of Ho Chi Minh City, No. 12 Nguyen Van Bao Street, Go Vap District, Ho Chi Minh City, Vietnam Industrial University of Ho Chi Minh City Ho Chi Minh City Vietnam

**Keywords:** Araceae, endemic, new species, *Typhoniumobtusum*, Vietnam

## Abstract

*Typhoniumobtusum* is described as a new species endemic to Vietnam. It is unique in the genus in having an oblong-elliptic spathe limb with an obtuse apex and yellowish-greenish filiform staminodes with a down-curved acumen. The ecology, distribution and assessment of the conservation status of the new taxon, as well as a key to all known *Typhonium* species in Vietnam, are provided.

## ﻿Introduction

The genus *Typhonium* (Schott 1829) of the Araceae is estimated to have about 80 to 100 species distributed over the world (Sriboonma et al. 1994; Hetterscheid and Boyce 2000; Boyce et al. 2012; Hetterscheid 2013; Low et al. 2021; POWO 2024). A latest checklist of 70 accepted *Typhonium* species names is provided online (POWO 2024). Indochina was proved to be the centre of *Typhonium* diversity with about 40 species described (Low et al. 2021; Pham et al. 2023). The genus was revised several times for Vietnam (Gagnepain 1942a, b; Pham-Hoang 1993, 2003; Nguyen and Vu 2004; Nguyen 2005, 2017). In fact, the last three decades have witnessed many new discoveries which make the total number of *Typhonium* in the country to be 23 (Nguyen and Croat 1997; Hetterscheid and Boyce 2000; Hetterscheid and Nguyen 2001; Nguyen 2005, 2008; Nguyen and Croat 2010; Hetterscheid 2013; Luu et al. 2017; Van et al. 2017, 2021; Nguyen et al. 2021, 2022a, b; Nguyen-Phi et al. 2023; Pham et al. 2023; Serebryanyi et al. 2023).

As part of ongoing study of *Typhonium* in Vietnam, we have collected several putatively new taxa, one of which was found in Phu Yen Province, central Vietnam. At the first glance, the plant looks like *T.rhizomatosum* A.Galloway and P.Schmidt (Galloway 2012) and *T.cordifolium* S.Y.Hu (Hu 1968) as all of them share the following in common: the general appearance of leaves and spathe limbs and the structure of inflorescences (Nguyen et al. 2022b; Nguyen-Phi et al. 2023). However, after our careful examination of its morphological characteristics, it turns out that our plant is, indeed, a new species that we describe here, based on living collections.

## ﻿Material and methods

The studied material was collected from Phu Yen Province, Central Vietnam. Specimens were sampled and processed using methods guided by the Royal Botanic Gardens, Kew (Bridson and Forman 1999); the herbarium acronyms follow Thiers (2024). Detailed photographs and description of taxonomically important characters of the new species were taken of fresh materials in the field using a digital camera. Taxonomic identification was based on morphological vegetative and reproductive characters following the aforementioned literature.

## ﻿Taxonomic treatment

### ﻿ Typhonium obtusum

Taxon classificationPlantaeAlismatalesAraceae

Luu, X.B.Nguyen-Le & H.C.Nguyen sp. nov.

309C8BB1-186C-5314-A621-6D06D1C3E78C

urn:lsid:ipni.org:names:77336459-1

Fig. 1

#### Type.

Vietnam. Phu Yen Province, Tay Hoa District, Hoa Thinh Ward; 12°54'30.1"N, 109°14'15.1"E, 30 m elevation; 5 August 2015; Nguyen Le Xuan Bach & Nguyen Hieu Cuong *PY495-505* (holotype SGN!; isotypes SGN!, PHH!).

#### Diagnosis.

*Typhoniumobtusum* is morphologically similar to *T.rhizomatosum* and *T.cordifolium* in having ovate leaf blades with cordate base, oblong and elongate spathe limb as long as the spadix and an elongated fusiform spadix appendix. However, the novel taxon can be distinguished from *T.rhizomatosum* by its subcylindrical (vs. globose to subglobose) tubers without (vs. with) rhizomatous offsets, stipitate and fusiform (vs. sessile and conical) spadix appendix) and oblong-elliptic (vs. narrowly triangular ovate) spathe limb and from *T.cordifolium* by its subcylindrical (vs. subglobose) tubers, leaves without (vs. with) adventitious buds, oblong-elliptic (vs. narrowly triangular ovate) spathe limb and stipitate and fusiform (vs. sessile and cylindrical) spadix appendix.

**Figure 1.**
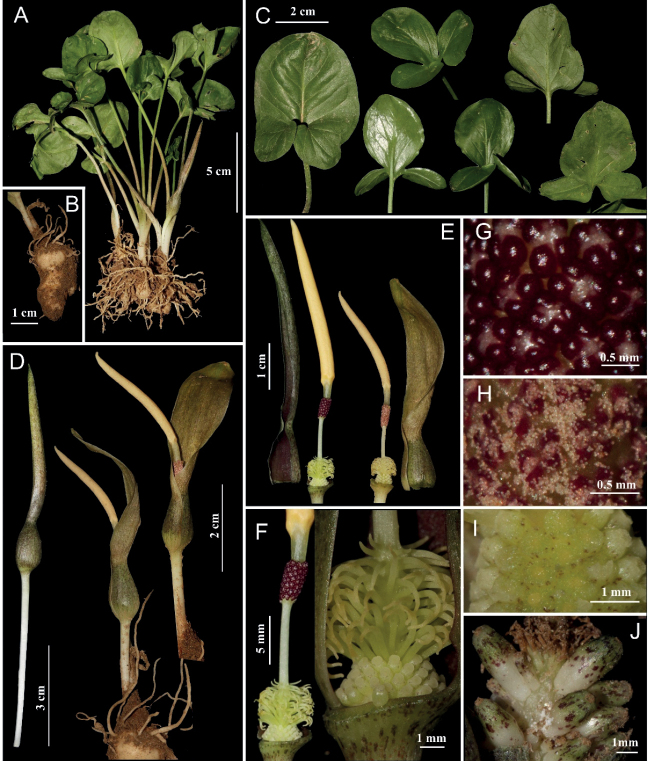
*Typhoniumobtusum***A** whole plants **B** stem **C** different forms of leaf blade **D** inflorescence **E** spathe and spadix **F** male, sterile and female zones **G** stamens **H** thecae, opened **I** pistils, cross-dissection **J** fruits.

#### Description.

Herbs, seasonally dormant, 10–17 cm tall; tuber underground, subcylindrical, fleshy, 2–2.5 cm long, 1–1.5 cm in diameter, with many filiform roots. ***Leaves*** 2–3 together. ***Petiole*** smooth, slender, 5–9 cm long, 1.8–2.2 mm in diameter, white to brown at base, green towards the apex. ***Leaf blade*** entire or trilobate, strongly cordate, glabrous, adaxially green, abaxially lighter green, concave, 3.2–4.7 cm long, 3–4 cm wide; entire leaf blade ovate, rounded at apex, venation pinnate with abaxially prominent midrib, lateral veins 5–8, brochiododromous, collective veins at 2–5 mm from margin; anterior lobes ovate, broadly elliptic to obovate, 2.5–3.7 cm long, 2.3–3.0 cm wide, obtuse to rounded at apex with a minute mucro, mid-rib abaxially prominent, lateral veins 4–6, brochiododromous, collective veins at 1–2 mm from margin; lateral lobes elliptic to ovate, 2.2–3.1 cm long, 0.6–1.6 cm wide, obtuse at apex, oblique at base. ***Inflorescences*** solitary; ***peduncle*** white, 2.5–6 cm long, ca. 4 mm in diameter; ***spathe*** 5–6 cm long, tube and limb separated by a constriction, outside green-brown, inside purple-brown; ***spathe tube*** ovoid, 1.1–1.3 cm long, 7–8 mm in diameter; ***spathe limb*** oblong-elliptic, 4–4.5 cm long, 1.1–1.3 cm wide, obtuse at apex, hardly open at base at anthesis; ***spadix*** equal or slightly shorter than spathe, sessile; ***female part*** conical, 1.6–2 mm long, 3.9–4.3 mm in diameter at the base, 2–3 mm in diameter at the top, with 3–4 rows of crowded pistils; ***ovary*** obovate, ca. 1 mm long, 0.6–0.8 mm in diameter, yellowish-greenish, with purple spots, unilocular, uniovulate placentation, transparently white; ***ovule*** light yellow, on a basal placenta and hold obliquely on a funicle; ***stigma*** sessile, disciform, 0.3–0.5 mm in diam., 0.1–0.2 mm high, light yellow, penicillate; ***interstice*** 1–1.3 cm long, lower part ca. 4 mm densely covered with staminodes, upper part naked, smooth, white to yellowish; ***staminodes*** filiform, subulate, 1–3 mm long, 0.4 mm in diameter, apically curved downward, yellowish-greenish; ***male part*** cylindrical, ca. 4.5 mm long, ca. 2.5 mm in diameter; ***stamens*** free, sparsely to (mostly) densely arranged; ***thecae*** globular, ca. 0.3 mm in diameter, dark purple, opening by apical slit; ***pollens*** translucent white; ***appendix*** stipitate, elongated fusiform, pale yellowish, 2.5–3.9 cm long, 2–3 mm in diameter, stipe ca. 3.5 mm long, pale yellow. ***Fruits*** ovoid to capsule-shaped, 3–4 mm long, 1.5–1.8 mm in diameter, white at base, green towards the apex with many dark purple spots.

#### Etymology.

The species is named for the obtuse apex of its spathe limb.

#### Vernacular name.

Bán hạ mo tù (Vietnamese); Obtuse-spathed typhonium (English, here proposed).

#### Ecological notes.

The new species was found growing in clumps on basalt soils in open places of rural farms. It appears in September to December and becomes dormant in January to August. Flowering and fruiting were seen in August.

#### Distribution.

*Typhoniumobtusum* has been recorded only from the type locality.

#### Conservation status.

Data Deficient (DD) (IUCN Standards and Petitions Subcommittee 2022). The new species has, so far, been found in one location and further inventory should be employed for a certain assessment.

#### Taxonomic notes.

The key morphological similarities and differences of *T.obtusum* versus *T.rhizomatosum* and *T.cordifolium* are presented in the diagnosis. In addition, the two latter are different from the new species by their unique characters in the genus: *T.cordifolium* with adventitious buds appearing at the mature leaf blade apex or sometimes at the top of the sheath, while *T.rhizomatosum* often forms large colonies thanks to its rhizomatous offsettings (Murata et al. 2010; Boyce et al. 2012; Nguyen-Phi et al. 2023).

The new species may be morphologically close to *T.hayatae* Sribonnma & J.Murata (Sriboonma et al. 1994), *T.inopinatum* Prain (King and Prain 1898), *T.medusae* Hett. & Sookch. (Hetterscheid et al. 2001) and *T.varians* Hett. & Sookch. (Hetterscheid et al. 2001), as they have a short spathe limb and similar general shape and structure of the spadix and filiform staminodes. However, these species are easily distinguishable from the new taxon as they have elongate conical, sessile or shortly stipitate spadix appendix with truncate base. Furthermore, *T.hayatae* has globose tuber, long spadix appendix (6–14 cm) and spathe limb (12–26 cm); *T.inopinatum* has globose tuber, ovate-lanceolate spathe limb that is shorter than the spadix appendix and yellow staminodes; *T.medusae* has depressed tuber, velvety petioles, hairy leaf blades, orbicular spathe limb, pale yellow or cream staminodes and elongate conical appendix; *T.varians* has depressed tuber, dark grey spadix appendix 4.5–9 cm long, triangular ovate spathe limb of 7–14 cm length and 4–7.5 cm in diameter and pale pink anthers. Their different morphological characters are summarised in Table 1.

**Table 1.** Morphological differences between *Typhoniumobtusum* and close species.

	*T.obtusum*	*T.hayatae*	*T.inopinatum*	*T.medusae*	*T.varians*	*T.rhizomatosum*	*T.cordifolium*
**Tuber**	subcylindrical, without rhizomatous offsets	globose, without rhizomatous offsets	subcylindrical, globose, without rhizomatous offsets	depressed, without rhizomatous offsets	depressed, without rhizomatous offsets	globose to subglobose, with rhizomatous offsets	subglobose, without rhizomatous offsets
**Leaf blade**	ovate, smooth	–	ovate to triangular or hastate, smooth	broadly triangular, 10 cm long, 14 cm wide, hairy	broadly triangular, more or less distinctly trilobate to subpentalobate, smooth	ovate to elliptical-ovate, smooth	narrowly ovate elliptic to narrowly elliptic, acuminate, with a bulbil when mature
**Spadix appendix**	2.5–3.9 cm long, 0.2–0.3 cm in diameter, pale yellowish, fusiform, stipe ca. 0.35 cm long, base gradually narrowing	6–14 cm long, 0.5–1.5 cm in diameter, conical, stipe short, base truncate	4–6 cm long, 0.4–0.5 cm in diameter, yellow, yellowish-brown, elongate conical, subsessile, base truncate	1 cm long, 0.3 cm in diameter, reddish-brown, elongate conical, stipe 0.15 cm long, base truncate	4.5–9 cm long, to 1 cm in diameter, dark grey, elongate conical, stipe 0.5 cm long, base truncate	8 cm long, 0.35 cm in diameter, beige, conical, sessile, base truncate	3.6–7.7 cm long, 0.1–0.2 cm in diameter, brick orange, cylindrical, sessile, base gradually narrowing
**Spathe tube**	ovoid, 1.1–1.3 cm long, 0.7–0.8 cm in diameter, green with brown dots	oblong-ovoid 2.5–4.5 cm long, 1–2 cm wide, dark brownish-purple	ovoid, 0.8‒1.5 cm long, ca. 1 cm in diameter, green	ovoid, 1.8–2 cm long, 1.2–1.5 cm in diameter, white with pinkish or purplish-brown flushing	ovoid, ca. 2.5 cm long, ca. 1.5 cm in diameter, glossy green	subglobose, 1 cm long, 0.8 cm in diameter, outside bright pale green, inside as outside, but with pale pink flush	ovoid, 1.2 cm long, 1 cm in diameter, light brown-green outside, brown or reddish-brown inside
**Spathe limb**	oblong-elliptic, 4–4.5 cm long, 1.1–1.3 cm wide, green with brown mottling	widely ovate in lower part, narrowly triangular in upper part, 12–26 cm long, 7.5–12 (–15) cm wide, dark brownish-purple	narrowly ovate to lanceolate, 5.5–7 cm long, 1.2–2 cm wide, basally brownish, apically green	orbicular, 2.7–3 cm long, 3 cm wide, whitish-greenish with a dense pinkish-brownish or brownish mottling	triangular ovate, 7–14 cm long, 4–7.5 cm wide, dark green flushed with dirty brown	narrowly triangular ovate, 7–9.5 cm long, 0.9–1.5 cm wide, bright pale green with brown longitudinal veins	narrowly triangular ovate, 5–9 cm long, to 3 cm wide, light brown-green
**Staminode**	slender filiform, decurved, yellowish-greenish	unknown	filiform, horizontally spread and slightly curved, yellow	subulate, upper ones straight, lower ones variously curved, mostly downwards, pale yellow or cream	subulate, upper ones straight, lower ones strongly curved downward, pale yellow	cylindrical, mostly perpendicular to axis, creamy white	cylindrical, spreading, dark yellow
**Ovary**	obovoid, yellowish-greenish	unknown	ellipsoid, yellowish-greenish	elongate, tapering to the base, basal half white, upper part spotted reddish-pink	more or less ellipsoid, pale green	elongate obovate, creamy white	elongate, white
**Stigma**	light yellow	unknown	yellow	reddish-pink	dirty whitish-greyish	creamy white	white
**Distribution**	C. Vietnam	S. Vietnam	N. and Central India to Thailand	C. Thailand	N. Thailand	Thailand	Thailand, Myanmar, S. Vietnam

##### ﻿Key to the 24 presently known Vietnamese species of *Typhonium*

**Table:** 

1	Sterile interstice of spadix entirely covered with staminodes	***T.flagelliforme***
–	Only base of sterile interstice of spadix covered with staminodes	**2**
2	Leaves perfectly trifoliolate	**3**
–	Not as above	**4**
3	Plant with 3 leaves; appendix stipitate; female section of spadix with 5–6 rows of pistils	***T.thatsonense***
–	Plant with 1 leaf; appendix sessile; female section of spadix with 2–3 rows of pistils	***T.hangiae***
4	Inflorescence appearing before the leaves	**5**
–	Inflorescence appearing together with the leaves	**6**
5	Spathe 6–9 cm long; staminodes ca. 6 mm long	***T.penicillatum***
–	Spathe 14–30 cm long; staminodes ≤ 3 mm long	***T.hayatae***
6	Spathe limb elongate, narrowly lanceolate-triangular	**7**
–	Spathe limb wide, oblong-elliptic, ovate to lanceolate	**15**
7	Staminodes red with a light yellow acumen	**8**
–	Staminodes unicolourful	**9**
8	Spathe tube globose, to 1.5 cm long; staminodes 5 mm long, clavate	***T.bachmaense***
–	Spathe tube oblong or cylindrical, 2 cm long; staminodes 12 mm long, acute	***T.kbangense***
9	Spathe limb white	***T.praelongum***
–	Not as above	**10**
10	Spathe limb corrugated	***T.corrugatum***
–	Not as above	**11**
11	Leaf 3-lobed	**12**
–	Not as above	**13**
12	Stigma funnel-shaped and lobed	***T.stigmatilobatum***
–	Stigma disciform and unlobed	***T.huense***
13	Leaves with 7 leaflets, leaflets linear to or linear-lanceolate	***T.lineare***
–	Not as above	**14**
14	Spathe limb much shorter than spadix appendix	***T.vermiforme***
–	Spathe limb as long as spadix appendix	***T.dongnaiense***
15	Spadix longer than spathe	**16**
–	Spadix as long as or shorter than spathe	**18**
16	Staminodes folded 180° apically	***T.phuocbinhense***
–	Staminodes straight up to parallel to axis	**17**
17	Male zone cylindrical, staminodes cylindrical to conical	***T.khonkaenensis***
–	Male zone subglobose, staminodes clavate	***T.acetosella***
18	Leaves developing bulbils at the top and/or the base, upper surface ften grey variegated	***T.cordifolium***
–	Not as above	**19**
19	Spathe limb very strongly circinnately recoiled over the entire length	***T.circinnatum***
–	Not as above	**20**
20	Tuber producing rhizomatous offsets about 5 cm apart	***T.rhizomatosum***
–	Not as above	**21**
21	Staminodes ≤ 3 mm long	**22**
–	Staminodes > 5 mm long	**23**
22	Spathe tube above the ground; spathe limb oblong-elliptic, with obtuse apex	***T.obtusum***
–	Spathe tube underground; spathe limb triangular ovate, with acute apex	***T.vietnamense***
23	Staminodes red with a light yellow acumen, upward straight or slightly curved	***T.blumei***
–	Staminodes whitish, curly	***T.trilobatum***

## Supplementary Material

XML Treatment for Typhonium obtusum

## ﻿Additional information

### Author ORCIDs

Hong Truong Luu https://orcid.org/0000-0002-7036-7081

Nga Nguyen-Phi https://orcid.org/0000-0001-7429-3641

Quoc Dat Nguyen https://orcid.org/0000-0002-3687-1567

Hieu Cuong Nguyen https://orcid.org/0000-0001-8707-2944

Hong Thien Van https://orcid.org/0000-0003-0151-5068

Xuan Bach Nguyen-Le https://orcid.org/0000-0001-8391-338X

### Data availability

All of the data that support the findings of this study are available in the main text.
